# A proteomics approach reveals molecular manipulators of distinct cellular processes in the salivary glands of *Glossina m. morsitans* in response to *Trypanosoma b. brucei* infections

**DOI:** 10.1186/s13071-016-1714-z

**Published:** 2016-08-02

**Authors:** Henry M. Kariithi, Sjef Boeren, Edwin K. Murungi, Just M. Vlak, Adly M. M. Abd-Alla

**Affiliations:** 1 Biotechnology Research Institute, Kenya Agricultural and Livestock Research Organization, P.O Box 57811, 00200 Kaptagat Rd, Loresho, Nairobi Kenya; 2 Laboratory of Biochemistry, Wageningen University, Dreijenlaan 3, 6703 HA Wageningen, The Netherlands; 3 Department of Biochemistry and Molecular Biology, Egerton University, P.O. Box 536, 20115 Njoro, Kenya; 4 Laboratory of Virology, Wageningen University, Droevendaalsesteeg 1, 6708 PB Wageningen, The Netherlands; 5 Insect Pest Control Laboratories, Joint FAO/IAEA Division of Nuclear Techniques in Food and Agriculture, International Atomic Energy Agency, Wagrammer Straße 5, Vienna, Austria

**Keywords:** LC-MS/MS, Protein-protein interaction, Metacyclic trypomastigotes, Metacyclogenesis, Vector competence, Trypanosome refractoriness

## Abstract

**Background:**

*Glossina m. morsitans* is the primary vector of the *Trypanosoma brucei* group, one of the causative agents of African trypanosomoses. The parasites undergo metacyclogenesis, i.e. transformation into the mammalian-infective metacyclic trypomastigote (MT) parasites, in the salivary glands (SGs) of the tsetse vector. Since the MT-parasites are largely uncultivable in vitro, information on the molecular processes that facilitate metacyclogenesis is scanty.

**Methods:**

To bridge this knowledge gap, we employed tandem mass spectrometry to investigate protein expression modulations in parasitized (*T. b. brucei*-infected) and unparasitized SGs of *G. m. morsitans.* We annotated the identified proteins into gene ontologies and mapped the up- and downregulated proteins within protein-protein interaction (PPI) networks.

**Results:**

We identified 361 host proteins, of which 76.6 % (*n* = 276) and 22.3 % (*n* = 81) were up- and downregulated, respectively, in parasitized SGs compared to unparasitized SGs. Whilst 32 proteins were significantly upregulated (> 10-fold), only salivary secreted adenosine was significantly downregulated. Amongst the significantly upregulated proteins, there were proteins associated with blood feeding, immunity, cellular proliferation, homeostasis, cytoskeletal traffic and regulation of protein turnover. The significantly upregulated proteins formed major hubs in the PPI network including key regulators of the Ras/MAPK and Ca^2+^/cAMP signaling pathways, ubiquitin-proteasome system and mitochondrial respiratory chain. Moreover, we identified 158 trypanosome-specific proteins, notable of which were proteins in the families of the GPI-anchored surface glycoproteins, kinetoplastid calpains, peroxiredoxins, retrotransposon host spot multigene and molecular chaperones. Whilst immune-related trypanosome proteins were over-represented, membrane transporters and proteins involved in translation repression (e.g. ribosomal proteins) were under-represented, potentially reminiscent of the growth-arrested MT-parasites.

**Conclusions:**

Our data implicate the significantly upregulated proteins as manipulators of diverse cellular processes in response to *T. b. brucei* infection, potentially to prepare the MT-parasites for invasion and evasion of the mammalian host immune defences. We discuss potential strategies to exploit our findings in enhancement of trypanosome refractoriness or reduce the vector competence of the tsetse vector.

**Electronic supplementary material:**

The online version of this article (doi:10.1186/s13071-016-1714-z) contains supplementary material, which is available to authorized users.

## Background

*Trypanosoma brucei* group causes African trypanosomoses, a group of neglected zoonotic tropical diseases endemic in 37 sub-Saharan African countries [1], against which there are no effective vaccines or drugs [2, 3]. Amongst the medically and agriculturally important tsetse fly species, *Glossina morsitans morsitans* is highly significant in the savannah woodlands and is the primary vector of the species of *T. brucei* group [4].

In order to complete their complex developmental cycles in the tsetse vector, trypanosomes face two critical replicative stage barriers: colonization of the midguts and establishment in the salivary glands (SGs). The pathway followed by the parasites between these two independent multi-replicative stages is time-dependent, irreversibly transient and eventually influences metacyclogenesis in the SGs (differentiation into the mammalian-infective metacyclic trypomastigote (MT) parasites) [5–9]. Critical replicative events occur ~3 days post-infective blood meal when only a small proportion (≤ 10 %) of the parasites is able to pass through the midgut barrier [8]. Upon establishment of infection, parasite numbers per fly gut remain remarkably constant [6, 8]. However, since the SG-derived MT-parasites are uncultivable in vitro, molecular mechanisms that promote metacyclogenesis remain to be investigated.

Whilst the SGs determine success of metacyclogenesis, there is limited knowledge on how trypanosomes adapt to and evade the host defence responses in the SGs [10]. It is however generally known that trypanosomes modulate SGs microenvironment [11], and that factors such as parasite genotypes, midgut antioxidant status, lectins and environmental stimuli influence parasite maturation [12, 13]. Most trypanosome research has focussed on the bloodstream and/or in vitro cultured procyclic forms, thus creating a knowledge gap with regard to metacyclogenesis. The availability of complete genomes of *G. morsitans* [14] and *T. brucei* [15] makes it possible to identify proteins involved in the mechanisms that facilitate metacyclogenesis. Such proteins are ideal candidates to develop improved strategies for tsetse and trypanosomosis control, especially via the sterile insect technique (SIT) programs [16], which have so far been employed in areas without active disease transmission [2].

Vector competence is one of the key pillars in the SIT programs, which involves mass release of sterile males into wild populations of the target species. More importantly, the sexually sterilized males are still competent trypanosome vectors, thus increasing risks of disease transmission when millions of sterile males are released into trypanosome-infested areas. Attempts have been previously made to make the sterile males vector incompetent via drug-supplemented blood meals, an approach now known to be inefficient [17]. Thus, alternative and/or complementary approaches are necessary, especially with the risk of trypanosomes developing resistance to the trypanocidal drug supplements. In this regard, metacyclogenesis and transmission of the mammalian-infective MT-parasites potentially represent vulnerable and attractive intervention points to enhance the natural trypanosome refractoriness or reduce the vectorial competence of the sterile males used in the SIT campaigns.

We hypothesized that *T. b. brucei* manipulates expression of proteins involved in pathways that specifically prepare the MT-parasites for successful transmission to and infection of susceptible mammalian hosts. To test this hypothesis, we employed tandem mass spectrometry (LC-MS/MS) to determine *T. b. brucei*-induced protein expression modulations in parasitized SGs of *G. m. morsitans* compared to unparasitized SGs. We also aimed at highlighting major metacyclic *T. b. brucei*-specific proteins, which are potentially critical for parasite survival in the SGs and transmission to susceptible mammalian hosts. We discuss our findings from the perspective of potential approaches to enhance trypanosome refractoriness in tsetse as an anti-vector strategy against African trypanosomosis.

## Methods

### Tsetse flies and parasites infections

Male *G. m. morsitans* were obtained from the Institute of Tropical Medicine (Antwerp, Belgium) and infected with a highly transmissible *T. b. brucei* strain (EATRO 1125 AnTaR 1) [11]. Male flies were used because they mature significantly more midgut infections than females [5]. For the infections, teneral flies (24–48 h post-adult eclosion) received their first blood meals supplemented with ~12 μg of parasites/fly [11]. Fully-engorged flies were selected, reared for 28 days post-infection (dpi) in controlled insectaria (65 % relative humidity; 26 °C) and subsequently fed on clean (trypanosome-free) defibrinated horse blood (in vitro; 2–3 times/week) [18]. Control flies were prepared from males of the same batch, age and feeding history as the parasitized flies. The control flies were maintained on clean blood meals and handled as described for their parasitized counterparts. All assays consisted of biological triplicates (20 flies per group) in small holding cells (3.5 cm in diameter × 6 cm height).

### Parasite scoring and SG dissections

Forty-eight h after the last blood meal, the flies were scored for the presence of the MT-parasites in the SGs using phase-contrast microscopy (×400) as previously described [11]. Briefly, the SGs were considered parasitized if stuffed with trypanosomes (i.e. the fly’s spit full of the parasites). Fly’s spit (from the control group) completely devoid of trypanosomes were considered unparasitized. For mass spectrometry, 10 intact pairs of SGs were selected from each of the replicated fly groups and immediately preserved at -20 °C in 150 μl sterile saline supplemented with complete protease inhibitor cocktail (Roche Diagnostics, Mannheim, Germany).

### SG extracts preparation and SDS-PAGE

SGs were individually homogenized using a glass/Teflon homogenizer and ultra-sonicated (Sonifier cell disruptor, Branson Instruments Co., Stanford, Connecticut, USA). Homogenates were freeze-thawed and clarified three times by centrifugation (7500× *g*; 10 min; 4 °C) to completely remove cell debris. Supernatants were pooled and proteins quantified using the standard BCA method (Bio-Rad, Hercules, California, USA). Equal protein quantities (600 ng) were resolved in 12 % SDS-PAGE gels and stained (CBB stain; NuPAGE Novex; Invitrogen Life Technologies, Carlsbad, California, USA). The middle sections of entire gel lanes were longitudinally excised (from top to bottom), divided into five equal fractions (covering the entire gel lanes) and cut into small pieces (~1 mm^3^) as previously described [19].

### LC-MS/MS measurements and protein identification

Tryptic peptides for subsequent LC-MS/MS measurements were prepared as previously described [19]. Briefly, the gel pieces were washed with 50 mM ammonium bicarbonate (ABC) buffer and ABC buffer/50 % acetonitrile (ACN) and proteins reduced and alkylated using dithiothreitol and iodoacetamide. Gels were washed with ABC/ABC-ACN buffer, followed by in-gel trypsin digestions and LC-MS/MS measurements [20]. Proteins were identified by searching the MS/MS spectra (using MaxQuant/Andromeda [21, 22]) against *G. m. morsitans* and *T. brucei* databases (downloaded from UniProt), a decoy database (constructed by reversing all the protein sequences) and a database of common contaminants (available from MaxQuant). MaxQuant search parameters included fixed carbamidomethylation (C), oxidation (M), acetylation and deamidation (N and Q). Two peptides (at least one unique and unmodified) matching the same protein were required for protein identification at a maximum false discovery rate (FDR) of ≤ 0.01. The unique identifiers of the proteins downloaded from the UniProt databases were used to identify and classify the LC-MS/MS peptides as specific to the host (*G. m. morsitans*) and the parasite (*T. b. brucei*). LC-MS/MS peptide hits to the decoy database and hits with modified peptides only were deleted from the final list of protein groups.

### Protein quantification and normalization

Unique and ‘razor’ (non-unique) peptides were used for peptide assignments and protein quantification [21, 23]. For each of the above-described five gel fractions, peptides were matched across different MS/MS runs based on mass and retention time (‘match between runs’ of 2 min). Label-free quantification (LFQ) was enabled. To minimize technical variations and to easily compare abundances of the same proteins (parasitized *vs* unparasitized), Log_10_ normalized LFQ was used across the biological triplicates. To compare levels of different proteins from the same samples (parasitized and unparasitized), Log_10_ iBAQ (intensity-based absolute quantitation) was used [24]. To determine up- and downregulated proteins (parasitized *vs* unparasitized SGs), *t*-tests were performed on Log_10_ LFQ using the MaxQuant’s Perseus module. Proteins were considered to be up- or downregulated when their Log_10_ iBAQ ratios (parasitized *vs* unparasitized) were larger or smaller than zero, respectively, and significantly upregulated when the FDR was ≤ 0.05.

### Gene ontology (GO) annotations and PPI network analyses of upregulated SG proteins

Blast2GO v. 3.2 [25] was used to classify the identified proteins along three biological aspects, i.e. biological process (BP), molecular function (MF) and cellular component (CC) Gene Ontology (GO) terms. It should be noted that the GO terms are descriptions of the different protein functional classes and how they relate to each other. To provide a broader overview of the ontologies, the GO classes were grouped into GO-slim terms using CateGOrizer [26]. To correctly place the significantly upregulated proteins within signaling pathways and networks, computational predictions of protein-protein interactions (PPIs) were inferred using interolog mapping [27]. For this, human orthologs to the identified *G. m. morsitans* proteins were obtained from Ensembl [28] and used to generate an exhaustive list of possible interacting protein combinations using custom in-house Python scripts. Putative interactions for these combinations were then determined using FpClass [29] (probability ≥ 0.2) and mapped back onto the SG protein datasets obtained from the above-mentioned LC-MS/MS measurements. The resulting significant interactome was rendered in Cytoscape v3.30 [30].

## Results

### Parasitized SG proteome of *G. m. morsitans*

**Fig. 1:**
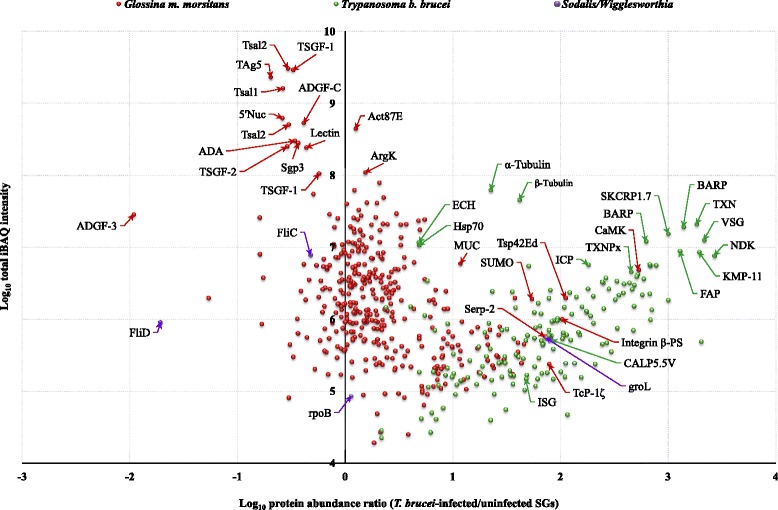
Distribution of proteins in parasitized *G. m. morsitans* SG proteome. Scatter plot distribution of the protein expression measurements for the identified *G. m. morsitans* proteins (*red*; *n =* 361), *Trypanosoma* proteins (*green*; *n =* 158) and bacterial endosymbiont proteins (*purple*; *n =* 4). Selections of the most notable proteins are named in the figure. See text for abbreviations

Analysis of the LC-MS/MS data resulted in 5469 and 4366 total and unique peptides, respectively. These peptides mapped to 874 protein groups. Removal of the common contaminants, applying extra filter steps such as removing single peptide hits (thereby decreasing FDR to below 0.01) and hits to the decoy database resulted in 523 non-redundant (nr) protein groups (Additional file 1: Table S1). Of these, 363 protein groups had specific peptide hits to the tsetse vector (*G. m. morsitans*) proteins (Additional file 1: Table S2) and 158 protein groups had specific peptide hits to the parasite (*T. b. brucei*) proteins (Additional file 1: Table S3). We also obtained peptide hits to four proteins specific to the bacterial endosymbionts *Wigglesworthia glossinidia* (*n* = 3) and *Sodalis glossinidius* (*n* = 1) (Additional file 1: Table S4). These symbiont proteins have been reported in the genome of *G. m. morsitans* [14]. It should be noted that there were no host and/or parasite and/or symbiont proteins that grouped together in one protein group. Figure 1 shows the abundance distribution of the proteins identified in parasitized SGs compared to unparasitized SGs.

Table 1 presents the topmost abundant 25 proteins, amongst which were the following blood feeding-associated proteins: tsetse salivary gland proteins-1 & 2 (Tsal1/2), tsetse salivary growth factors-1 & 2 (TSGF-1/2), salivary antigen-5-protein (TAg5), 5′-nucleotidase-related saliva protein (5′Nuc), adenine deaminase (ADA) and 5′-nucleotidase-related SG protein-3 (Sgp3). Also abundant were proteins related to cellular proliferation/differentiation (adenosine deaminase-related growth factors, muscle LIM protein at 84B), cytoskeletal proteins of the actin and myosin families, lectins, molecular chaperones (e.g. 14-3-3 protein zeta family chaperones) and enzymes (e.g. trehalose-6-phosphate synthase, isovaleryl-CoA dehydrogenase, and aspartate aminotransferase).

**Table 1:** Annotations of the most abundant host proteins detected in *Glossina m. morsitans* SGs. Proteins are listed from the most abundant in descending order

UniProt ID	Protein name	Mol. weight (kDa)	Sequence coverage (%)	Peptides	Log_10_ iBAQ	-Log *t*-test (*P*-value)	*t*-test ratios (infected *vs* unparasitized)	Description/functional roles in insects
D3TMW5	Tsetse salivary gland protein 2	43.955	87.9	50 [2]	9.48	2.67	-0.53 (↓)	Blood-feeding; immunogenic
D3TLK6	Tsetse salivary gland growth factor-1	56.59	78.9	66 [5]	9.46	3.60	-0.49 (↓)	Blood-feeding and anti-haemostasis
D3TQL1	Salivary antigen 5 variant	28.925	78.4	32 [18]	9.36	3.18	-0.69 (↓)	Blood-feeding and other extensive physiological roles
Q9NBA5	Tsetse salivary gland protein 1	45.613	87.0	45 [20]	9.21	2.16	-0.58 (↓)	Blood-feeding; immunogenic
D3TRV7	5′-nucleotidase-related (5′Nuc) saliva protein	62.064	67.2	37 [37]	8.80	3.06	-0.58 (↓)	Blood-feeding; downregulated in parasitized flies
D3TQW4	Adenosine deaminase-related growth factor C	62.2	65.6	39 [32]	8.73	1.96	-0.38 (↓)	Cell proliferation; non-immunogenic
Q9NBA4	Tsetse salivary gland protein 2	44.001	84.5	48 [9]	8.71	1.88	-0.52 (↓)	Involved in blood-feeding; immunogenic
D3TPT6	Actin 87E	41.831	72.1	25 [1]	8.65	0.21	0.10 (↑)	Overexpressed in hytrosavirus-infected tsetse
D3TKU2	Adenine deaminase	24.14	46.7	20 [2]	8.48	3.01	-0.47 (↓)	Blood-feeding; vector competence; cellular proliferation
D3TKU0	5′nucleotidase	100.2	27.7	30 [30]	8.45	1.42	-0.44 (↓)	Blood-feeding; downregulated in parasite-infected flies
Q9U7C5	Tsetse salivary gland growth factor-2	58.222	57.1	40 [40]	8.40	2.09	-0.54 (↓)	Blood-feeding and antihaemostasis; non-immunogenic
D3TR78	Lectin	19.762	63.3	16 [16]	8.39	2.09	-0.36 (↓)	Influence trypanosome establishment and maturation
D3TPN5	Arginine kinase	40.029	65.4	26 [25]	8.04	1.05	0.19 (↑)	Abundantly expressed in silkworms; insect homeostasis
Q9U7C6	Tsetse salivary gland growth factor-1	56.631	76.3	63 [2]	8.02	0.44	-0.24 (↓)	Blood-feeding and antihaemostasis; non-immunogenic
D3TQC9	Muscle LIM protein at 84B	10.077	61.3	6 [1]	7.90	1.46	0.32 (↑)	Muscle/epithelia differentiation in *Drosophila*
D3TQ00	Myosin heavy chain	87.317	67.1	68 [68]	7.79	0.09	0.08 (↑)	Overexpressed in hypertrophied tsetse SG
D3TRK1	Trehalose-6-phosphate synthase	31.361	57.2	18 [18]	7.74	2.14	-0.29 (↓)	Tsetse housekeeping gene involved in trehalose synthesis
D3TLM8	Multifunctional chaperone (14-3-3-ζ)	28.213	59.7	13 [11]	7.69	2.64	0.36 (↑)	Intracellular adaptor in diverse biological processes
D3TRW4	ATP synthase β	54.579	64.3	21 [20]	7.62	0.70	0.20 (↑)	Ion transporter
D3TN30	Cytochrome c2	11.768	54.6	7 [7]	7.62	1.13	0.29 (↑)	Essential mitochondrial respiratory chain component
D3TR28	Calponin	20.152	81.5	15 [15]	7.60	0.05	0.01 (↑)	Ca^2+^-binding protein; associated with wound-healing
D3TLI1	Troponin I	24.523	36.1	9 [9]	7.52	0.28	0.12 (↑)	An actin-binding protein
D3TQ27	Cofilin actin depolymerizing factor/Cofilin)	17.167	73.0	12 [12]	7.47	2.99	0.48 (↑)	Control of actin assembly in cells
D3TNV8	Elongation factor 1α	50.403	60.9	18 [13]	7.46	0.79	-0.15 (↓)	Translation elongation
D3TQW6	Salivary secreted adenosine	41.221	29.2	20 [2]	7.46	1.13	-1.96 (↓)	Non-immunogenic ADGF (also known as ADGF-3)

Except salivary secreted adenosine (significantly downregulated), all the other proteins shown in this table were insignificantly upregulated (*n* = 10) or downregulated (*n* = 15) in parasitized SGs compared to the unparasitized GGs. Upregulated and downregulated proteins are indicated by upward (↑) and downward (↓) arrows, respectively. The unique peptides for each of the proteins listed in the table are shown in square brackets in column 5

Amongst the least abundant proteins that we identified included dihydrolipoamide transacylase α-ketoacid dehydrogenase (DBT) complex proteins (for amino acid metabolism), GTP-binding proteins, Ras-related protein and clathrin adaptor complex proteins (for endocytotic trafficking), 26S proteasome regulatory complex proteins (for protein turnover), bifunctional ATP/sulfurylase-adenosine 5′-phosphosulfate (APS) kinase (for uptake/assimilation of inorganic sulphate) and translocon-associated (TRAP) complex proteins (for endoplasmic reticulum (ER)-targeting of nascent polypeptides) (see Additional file 1: Table S2).

The parasitisation of *G. m. morsitans* SGs appears not to have affected the expression of at least four host proteins, including bifunctional methylene-tetrahydrofolate dehydrogenase (MTHF-folD) (for thymidylate/methionine/purine synthesis) and 25-/28-kDa glutathione S-transferase 1 (GST1) proteins (for detoxification and lipophilic compound transport) (Additional file 1: Table S8).

### Preferentially expressed proteins in parasitized SGs and *T. b. brucei*-induced changes

Compared to the unparasitized SGs, 276 *G. m. morsitans* proteins were found to be upregulated in the parasitized SGs (Table S5); of these 32 proteins were significantly upregulated (Table 2). Topmost of the upregulated proteins included Ca^2+^/calmodulin-dependent protein kinase (CaMK), tetraspanin 42Ed (Tsp42Ed), β-integrin, stress-associated ER protein-2 (Serp-2), small ubiquitin-related modifier-3 (SUMO), a homolog to uracil-DNA degrading factor-like (UDE) protein and various subunits of the vacuolar ATPases and chaperonin containing t-complex polypeptide-1 (TcP-1). Others included amino acid metabolism-related proteins such as methylglutaconyl-CoA hydratase (AUH), glutamine synthetase (GS), δ-1-pyrroline-5-carboxylate dehydrogenase (P5CDH), aspartate aminotransferase (AspAT) and isovaleryl-CoA dehydrogenase (IVD).

**Table 2:** Annotation of 32 *Glossina*-specific proteins significantly upregulated in parasitized SG proteome of *Glossina m. morsitans* compared to the unparasitized SG proteome

UniProt ID	Protein name	LC-MS/MS measurements/quantification				Protein descriptions/functional annotation
		Mol. weight (kDa)	Sequence coverage (%)	Unique peptides	-Log *t*-test (*P-*value)	
D3TQ33	Ca^2+^/calmodulin-dependent protein kinase	24.064	58.1	11	5.14	Calcyphosin-like protein; regulation of ion transport
D3TMA1	Tetraspanin 42Ed	25.291	13.5	3	3.98	Acts as scaffold/anchor to specific cell membranes
D3TQS8	Integrin beta-PS	27.471	24.5	5	5.64	IGF-like repeat protein; cell adhesion to extracellular matrix proteins
H9TZT6	Stress-associated ER protein-2	42.284	17.6	4	4.21	ER stress
D3TQD5	Small ubiquitin-related modifier 3	10.328	35.2	3	1.49	Essential regulator of cellular processes (e.g. survival of stressed cells).
D3TMM3	Vacuolar H+−ATPase v0 sector subunit D	39.805	19.1	4	3.60	Cation trans-epithelia transport (SGs, labial glands; midguts; sensory sensilla)
D3TMK9	Chaperonin containing t-complex polypeptide-1ζ	58.314	13.7	5	4.20	TcP-1 family members are involved in the prevention of the aggregation of proteins unfolded by stress, or newly synthesized proteins
D3TM06	Chaperonin containing t-complex polypeptide-1θ	60.02	11.8	5	5.57	
D3TLV7	Chaperonin containing t-complex polypeptide-1ζ	59.292	10.9	4	5.46	
D3TLP9	Chaperonin containing t-complex polypeptide-1α	59.192	5.0	3	1.21	
D3TMQ1	Mitochondrial methylglutaconyl-CoA hydratase	31.879	19.1	4	4.18	Metabolism of branched-chain amino acids (e.g. leucine, isoleucine and valine)
D3TLC7	Isovaleryl-CoA dehydrogenase	46.634	5.7	2	3.84	
D3TNK0	Hypothetical conserved protein	37.228	10.8	4	4.70	70 % homologous to *D. melanogaster* uracil-DNA degrading factor-like protein (UDE); conformational integrity of DNA-protein complex machinery
D3TPX7	α-carboxylesterase αE7	65.575	21.8	11	1.44	Lipid metabolism in insects
D3TMQ8	24-kDa mitochondrial glutamine synthetase	44.028	4.2	2	2.03	Metabolism of glutamate (important product of ammonia detoxification)
D3TLS2	Mitochondrial NADH-ubiquinone oxidoreductase	26.829	13.2	3	3.08	Mitochondrial electron transport/transfer
D3TRJ5	Cytochrome b-c_1_ complex-7	13.551	28.8	3	0.92	
D3TPP1	Downstream of receptor kinase	24.434	13.7	3	3.19	Essential roles in immune responses
D3TMW2	Proteasome subunit beta type-4	30.601	7.7	2	4.76	Associates with polo-like kinase; increase 20S proteasome to proteolytic activity
D3TPR8	Translin	28.855	14.4	3	3.66	Various biological roles (e.g. control and distribution of nucleic acid metabolism)
D3TLJ6	Mitochondrial prohibitin-2	36.64	9.7	3	1.01	Conserved protein involved in biogenesis and maintenance of mitochondria
D3TMK2	Ras-related small GTPase, rho type	21.289	12.0	2	5.47	Molecular switches that govern various important cellular functions
D3TRZ7	Aspartate aminotransferase	45.969	21.9	6	1.10	A key enzyme in amino acid metabolism
D3TRZ8	Gamma-glutamyl hydrolase	43.565	4.5	2	5.92	Folate metabolism
D3TPR2	Myosin essential light chain	16.572	27.2	3	0.93	A structural component of the actomyosin cross-bridge
D3TRA4	Transaldolase	37.217	20.5	5	0.95	Provides a link between glycolytic and pentose phosphate pathways
D3TLJ8	Dihydrolipoamide S-acetyltransferase	55.469	8.2	3	1.14	Provides a link between glycolytic and TCA cycles
D3TNC5	Retrotransposon protein	28.099	17.4	3	2.87	Mobile element that transpose by reverse transcription
D3TP54	Actin-related protein 2/3 complex-3	20.502	15.8	2	3.31	Induction of actin polymerization during pathogen infection
D3TQ55	Hypothetical secreted protein	24.355	38	2	2.60	Homologous (97 %) to salivary secreted mucin; tsetse mouthpart lubricant
Q0QHK6	δ-1-pyrroline-5-carboxylate dehydrogenase	58.318	26.3	10	2.61	Amino acid (glutamate and proline) metabolism
D3TRY4	Cathepsin B-like cysteine proteinase	38.221	14.1	4	3.37	SG cell autophagic cell death

Several proteins associated with the ubiquitin-proteasome system (UPS) were also upregulated (Additional file 1: Tables S5 and S6), including ubiquitin C-terminal hydrolase (uCHL), ubiquitin-40S ribosomal protein S27a fusion protein (RpS27A), ubiquitin activating enzyme (uBA1), ubiquitin-protein ligase (E3) and various subunits of the proteasome regulatory complex proteins α-/β-types (PSMA/PSMB). According to our protein identification criteria, the repertoire of the downregulated proteins did not have any UPS-associated proteins.

Nine proteins known to be involved in *Glossina* immunity were upregulated. Among these were thioredoxin peroxidases (TrxP), alkyl hydroperoxide reductase (AhpC), Serp-2, cathepsin B-like cysteine proteinase (CatB), a truncated nitric oxide synthase (NOS) and transferrin. Of these, only Serp-2 and CatB were significantly upregulated (Additional file 1: Tables S5 and S6). Other putative immunity-related proteins included integrin-β, Tsp42Ed and adaptor protein downstream of receptor kinase (Drk), all of which were significantly upregulated. Top BLASTp (bitscore >75; E-value ≤1.0E-4) of these immunity-related proteins against the Insect Innate Immunity Database (IID) v 2.2.26 [31] resulted in significant homologies to immunity proteins that have been reported in the *Acyrthosiphon pisum* (pea aphid), *Drosophila melanogaster* (common fruit fly), *Apis mellifera* (western honeybee) and *Anopheles gambiae* (malaria mosquito) (Table 3).

**Table 3:** Top BLASTp scores of 18 immunity proteins detected in parasitized SGs of *G. m. morsitans*

UniProt ID	Protein name	Best Blast Match (description of homologies)					Pathway	Processes and roles in insect immunity
		Best match (species name)	% identity	Accession No.	Bits score	E-value		
Q8MX87	Transferrin	Transferrin-1; (*D. melanogaster*)	63	AAF48831.1	827	0.0	Cell cycle regulation	Pathogen-induced; iron metabolism; cellular homeostasis (prevents hydroxyl radical toxicity)
Q8IS37	Nitric oxide synthase	Nitric oxide synthase; (*A. pisum*)	48	XP_001946209.1	137	1e-34	IMD	Response to production of NO; inactivation of critical enzymes in energy metabolism and growth of parasites
H9TZT6^b^	Stress-associated ER protein-2	Serine protease inhibitor-4; (*D. melanogaster*)	43	NP_724511.2	310	3e-86	Toll	Pathogen recognition and apoptosis (activation of Toll pathway). Serpins are determinants for *Plasmodium* susceptibility and transmission in mosquitoes
Q2PQQ0	Serine protease inhibitor-4		52		382	1e-107		
Q694B0	Thioredoxin peroxidase-3	Thioredoxin-dependent peroxidase-1; (*An. gambiae*)	73	XP_310704.3	295	6e-82	Humoral response	Thioredoxin redox system provides primary defence lines in insects (oxidative stress); increase in oxidative stress limits parasite maturation; oxidative stress plays important roles in refractoriness of tsetse to trypanosome infection
D3TN04	Alkyl hydroperoxide reductase		74		298	1e-82		
Q694A5	Thioredoxin peroxidase-1	Thioredoxin-dependent peroxidase-2; (*An. gambiae*)	76	XP_308081.2	315	4e-88		
Q694A6	Thioredoxin peroxidase-2	Thioredoxin-dependent peroxidase-3; (*An. gambiae*)	73	XP_308336.4	341	1e-95		
D3TRY4^b^	Cathepsin B-like cysteine protease	Cathepsin L; (*D. melanogaster*)	24	AAF51924.1	59	2e-10	Cellular response	Upregulated in immune-stimulated *Drosophila* and *G. m. morsitans* (phagocytosis-mediated immunity)
D3TPP1^a,^ ^b^	Downstream of receptor kinase	Plenty of SH3-domain protein (POSH); (*D. melanogaster*)	31	NP_523776.1	89.7	7e-08	Toll; IMD; signalling	DrKs are important downstream regulator of tumour necrosis factor/ c-Jun N-terminal kinase (TNF/JNK) signaling. JNK activation and Relish induction are delayed and sustained in POSH-deficient flies
D3TQS8^a,^ ^b^	Integrin-β	No hits found					Cellular response	Tetraspanins regulate integrin activity; provides scaffold for phagocytosis-mediated insect immunity
D3TMA1^a,^ ^b^	Tetraspanin 42Ed	No hits found						
D3TMK2^b^	Ras-related small GTPase rho type	Ras-related C3 botulinum toxin substrate-1; (*D. melanogaster*)	70	NP_476950.1	271	9e-75	Cell cycle regulation	Rac/Rab GTPases are required for proper encapsulation (phagocytosis-mediated immunity); Rac1/2 GTPases are necessary for immune surveillance against pathogens and parasites in *Drosophila*
D3TMD3	Rab protein-14 (Rab-14)		27		67.8	2e-13		
D3TRT5	Rab protein-5 (Rab-5)		25		60.1	5e-11		
D3TSA8	Rab protein-7 (Rab-7)	Ras-related C3 botulinum toxin substrate-2; (*D. melanogaster*)	27	NP_648121.1	66.2	7e-13		
D3TP17	Ras-related GTPase	Ras-related protein; (*A. mellifera*)	25	XP_623951.1	66.6	5e-13		
D3TQN6	Ubiquitin protein ligase (also known as E3)	Bendless/ ubiquitin conjugating enzyme 13; (*D. melanogaster*)	33	ACZ95287.1	100	3e-23	Toll; IMD; signaling	Humoral immune response. Bendless mutants have inefficient IMD pathway induction

Protein sequences were blasted against the Insect Innate Immunity Database (IIID). Sixteen of these proteins have been reported to be upregulated in the midguts, fat bodies and SGs of different trypanosome-infected tsetse species. The remaining three proteins ^a^are implicated in the immunity of other insects (see citations in the manuscript text). All the 18 proteins were upregulated in parasitized SGs compared to unparasitized SGs; six proteins ^b^were significantly upregulated

### Gene ontology (GO) and PPI network analyses of parasitized SGs during *T. b. brucei* infection

**Fig. 2:**
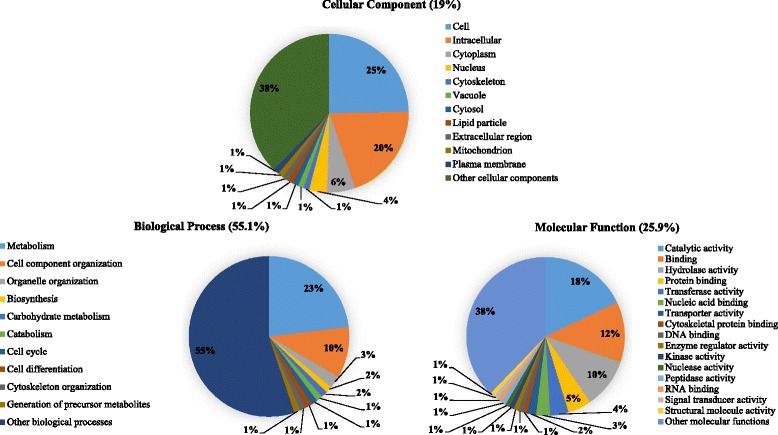
GO-term associations of significantly upregulated proteins in parasitized *G. m. morsitans* SG proteome. The GO classes were grouped into 127 Go-Slim terms associated with biological processes (55.1 %), molecular functions (25.9 %) and cellular component (19 %) ontologies

All of the significantly upregulated proteins were associated with at least one GO term for a total of 9323 term occurrences. Using the CateGOrizer, the GO terms were trimmed down and grouped into 127 Go-Slim terms associated with biological processes (BP; 55.1 %), molecular functions (MF; 25.9 %) and cellular components (CC; 19 %) ontologies (Fig. 2). Proteins involved in metabolism and cell- or organelle reorganization were present amongst the key BP ontologies (Additional file 1: Table S9), while the majority of MF ontologies represented proteins associated with catalytic, binding and hydrolase activities (Additional file 1: Table S10). Topmost in the CC ontology were proteins localized in the cellular, intracellular and cytoplasmic compartments of the SGs (Additional file 1: Table S11).

**Fig. 3:**
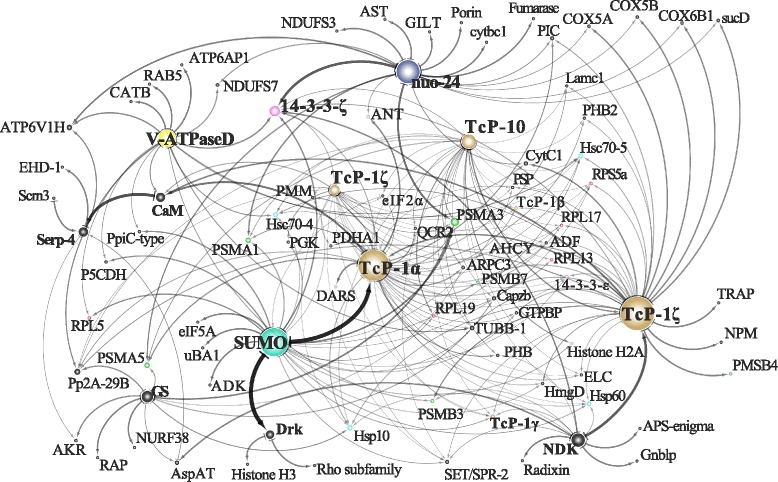
The most significant PPI network model for the upregulated proteins in parasitized SG proteome of *G. m. morsitans*. Highlighted are the main hubs formed by the proteins that were found to be significantly upregulated in the parasitized SGs. The PPI was visualized in Cytoscape. The significantly upregulated proteins SUMO, TcP-1, nuo-24, V-ATPase-D, Drk and GS occupy central positions in the PPI network

The predictions of protein-protein interaction network of the upregulated host proteins resulted in 10,861 putative interacting protein pairs, of which 225 pairs had significant interacting probabilities (Additional file 1: Table S12). A single high-scoring network was obtained consisting of 88 nodes, a clustering coefficient of 0.107 and an average number of neighbours of 5.0 (Fig. 3). Eighteen of the significantly upregulated proteins formed nine of the main PPI network hubs (Fig. 3). Seven of these proteins formed network nodes with the most edges including SUMO, TcP-1s, mitochondrial NADH-ubiquinone oxidoreductase (nuo-24), V-ATPase-D, Drk and GS.

### Expression of abundant proteins is downregulated in parasitized SGs

A total of 81 proteins were downregulated in parasitized SGs. However, only salivary secreted adenosine (ADA; previously annotated as adenosine deaminase-related growth factor-3; ADGF-3) was significantly downregulated (Additional file 1: Table S7). Despite their high abundance, the expression patterns of TAg5, 5′-Nuc, Tsal1/2, TSGF-1/2, ADA, ADGF-C, Spg3 and TRAP was downregulated. Further, > 20 % (*n* = 18) of the downregulated proteins were ribosomal protein (RP) components (Additional file 1: Table S7), including several proteins of the 40S and 60S subunits, glycine/glutamate-rich protein (Sgp1), proline-rich protein (Sgp2), mu-clathrin adaptor complex proteins, subunits of the vesicle coat complex II (COPII), ADGF-C and lectins.

### Expression of metacyclic trypomastigote-specific proteins

Table 4 presents major clusters of trypanosome-specific proteins identified in this study. Notable abundant proteins included the glycosylphosphatidyl inositol (GPI)-anchored proteins bloodstream stage alanine-rich proteins (BARPs) and variant/invariant surface glycoproteins (VSGs/ISGs), calpains/small kinetoplastid calpain-related proteins (CALPs/SKCRPs), retrotransposon hot spot proteins (RHS), kinetoplastid membrane protein-11 (KMP-11), tryparedoxins (TXNs) and membrane transporters. We also identified several trypanosome-specific RPs, with an over-representation of the 40S RP compared to the 60S RP families (Table 4). Also identified were paraflagellar rod proteins (PFRA and PFRC), flagellar attachment zone protein 1 (FAZ1), flagellar calcium-binding protein (Tb-44A) and C-terminal motor kinesin (Table 4). Here, it is important to note that FAZ and C-terminal motor kinesin are important in the adjustments of the flagellar positions/sizes [32], depending on the parasite life-cycle stages.

**Table 4:** Major clusters of *T. brucei* specific proteins identified in this study

Protein family	UniProt ID	Protein name	Functional roles
*Variant/invariant surface glycoproteins (VSG/ISG)*	Q26842	VSG	VSGs/ISGs are activated in the SGs; involved in immune evasion/resistance
	M4TB38	VSG 1228	
	M4SU87	VSG 725	
	Q57VX7	75 kDa ISG	
	B2ZWC6	ISG	
*Retrotransposon hot spot protein (RHS) multigene family*	Q8T9M3	RHS1a	RHSs are diverse and potentially rapidly evolving nuclear and perinuclear proteins in *T. brucei*. RHSs are located in the polymorphic subtelomic regions and may therefore confer selective advantages for the evasion of host immune responses through antigenic variations
	Q8T9M7	RHS2a	
	Q8WPS8	H25N7.12 protein (RHS4)	
	Q8T9M4	RHS6a	
	D0A6L8	Putative RHS	
	D7SGA2	RHS4	
	Q584N8	Putative RHS	
	Q585G9	RHS5a	
*Kinetoplastid calpain/small kinetoplastid calpain-related protein (CALP/SKCRP) protein family*	Q4GZ11	CALP1.1	CALPs are well-conserved and ubiquitously expressed in tissue-specific isoforms. They are involved in virulence and various physiological (cytoskeleton rearrangement, proliferation, cellular differentiation, interaction with host structures)
	C9ZIE8	CALP1.2	
	Q57WJ7	CALP5.5 V	
	C9ZT01	CALP7.1	
	Q387E1	CALP11.6	
	Q4GZ06	SKCRP1.4	
	C9ZIF2	SKCRP1.5	
	C9ZIF6	SKCRP1.7	
*Paraflagellar/paraxial rod (PRF) protein family*	C9ZVV0	69 kDa PFR-A	69-kDa and 73-kDa proteins are the major structural components of *T. brucei* flagellar; Important for parasite motility
	C9ZLC1	73 kDa PFR-C	
*Peroxiredoxin alkyl hydroperoxide reductase C (AhpC)-type family*	C9ZL57	Tryparedoxin	TXN/TXNPx are highly abundant in all life stages of *T. brucei*; Important in trypanosome metabolism (nucleotide synthesis); and oxidative defence (detoxification of hyperoxides)
	C9ZUX7	Tryparedoxin peroxidase	
	C9ZXT5	Tryparedoxin peroxidase	
*Bloodstream stage alanine-rich protein (BARP)*	C9ZZP8	BARP	BARPs are GPI-anchored proteins, which are important for cytokinesis; BARPs are proposed to form stage-specific coat for epimastigote forms of *T. brucei*
	O60946	BARP	
	Q38CW0	BARP	
	Q38CW1	BARP	
	C9ZZQ0	BARP	
*Molecular chaperones; heat shock proteins (Hsp) family*	C9ZL02	Putative Hsp20	Molecular chaperones are central players in various physiological processes such as protein folding and in maintenance of cellular homeostasis/survival under optimal growth conditions
	D0A349	Mitochondrial Hsp60	
	C9ZR44	Mitochondrial Hsp70	
	Q383E5	Hsp70	
	D0A4N5	Hsp83	
	D0A590	Putative Hsp	
*Membrane transporters*	Q388Z2	Plasma membrane ATPase (PMA1)	Involved in salvage of nutrients and other metabolites from the host
	C9ZK10	ATP synthase subunit beta (ATP5B)	
	Q581D7	Putative adenosine transporter 1 (ENT1)	
*Ribosomal proteins (RPs)*	D0A7E1	RPS5	Involved in the regulation of protein translation
	C9ZXI9	RPS7	
	O76223	RPS12	
	C9ZRH0	RPS14	
	D0A2S1	RPS18	
	C9ZZX2	RPL10a	
	C9ZYV4	RPL23	

Functional roles were inferred from available literature. Only a selection of variant/invariant surface glycoproteins (VSGs/ISGs) is shown in this table (see full list of the isoforms and/or variants in Additional file 1: Table S3)

Twenty-five of the parasite protein groups were of unknown functions (denoted as ‘uncharacterized proteins’ in Additional file 1: Table S3). Top-BLASTp analyses (bitscore >100; E-value ≤ 1.0E-6) of these proteins against the nr-NCBI protein database yielded eight hits to proteins with known functions in flagellate protozoans (Table 5). Six of these were hits to proteins reported in the recently sequenced genome of the African crocodilian trypanosome, *T. grayi* (vectored by *G. palpalis*), which is more closely related to *T. cruzi* than *T. brucei* [33]. Other homologies included proteins associated with antigenic variations (Pro-Glu/polymorphic GC-rich repeat (PE-PGRS) protein [34] and acyl-CoA-binding protein [35]), parasite proliferation (auxin-induced in root cultures 9 (AIR9)-like protein [36]) and the chemosensational intraflagellar transport, osmotic avoidance abnormal protei3 (OSM3)-like kinesin [37].

**Table 5:** BLASTp similarity scores for *T. b. brucei* uncharacterized proteins using Phylum Euglenozoa non-redundant NCBI database

UniProt ID	Length (aa)	Best BLASTp match (description of homologies)					Functional characterization; signature domains/motifs
		Homology hits (species name)	% identity	Accession No.	E-value	Bits score	
C9ZMR8	150	Flagellar associated protein; (*T. grayi*)	76	XP_009315587.1	4.00e-81	242	p25-alpha domain-protein; promote tubulin polymerization
C9ZJQ8	1488	Pro-Glu/polymorphic GC-rich repeat (PE-PGRS) protein; (*T. grayi*)	36	XP_009311393.1	1.00e-27	121	Antigenic variations
C9ZWF1	607	Calpain-like cysteine peptidase; (*T. grayi*)	66	XP_009312440.1	0.0	778	A ribonuclease inhibitor-like protein involved in cell cycle progression in parasites
Q57XH8	459	*T. brucei* (*s.l.*)-specific protein; (*T. b. gambiense*)	41	XP_011775378.1	5.00e-91	290	–
C9ZL20	483	Succinate dehydrogenase flavoprotein subunit; (*T. grayi*)	57	XP_009307889.1	0.0	528	Mediates protein-protein interactions/assembly of multi-protein complexes
C9ZU33	97	Acyl-CoA-binding protein-like protein 3; (*T. grayi*)	69	XP_009316294.1	1.00e-43	142	Supply of myristoyl-CoA to the fatty acid remodelling machinery of GPI biosynthesis in trypanosomes; antigenic variations
Q380Y7	1004	Auxin-induced in root cultures 9 (AIR9)-like protein; (*T. b. brucei*)	98	CBY84490.1	0.0	2028	Expressed in all life-cycle stages; essential for normal *T. brucei* proliferation in vitro
D0A668	462	Osmotic avoidance abnormal protein 3 (OSM3)-like kinesin; (*T. grayi*)	46	XP_009307651.1	2.00e-69	234	Intraflagellar (chemosensation) transport

Functional characterization of the proteins is detailed in the last column. All the proteins listed in this table are uncharacterized

## Discussion

A robust immune system in tsetse midguts makes the flies naturally *Trypanosoma*-refractory [38]. Following an infected blood meal, the absolute parasite numbers drastically drop at the midgut barrier (days 1–3), then proliferates (day 4) and stably colonizes the midguts where the established population reaches approximately 5 × 10^5^ trypanosomes [8, 39]. Trypanosomes then migrate to the ectoperitrophic space (day 5) [39], congregate within the proventriculus (days 6–8) and subsequently colonize and complete metacyclogenesis in the SGs (days 12–18) [8]. The MT-parasites then detach from the SG epithelium into the lumen and are uniquely adapted to infect and survive in susceptible mammalian host [32]. The entire process takes approximately 3–5 weeks [40], implying that at the time of the SG dissections (i.e. 28 dpi), MT-parasites were continuously produced.

### *Trypanosoma b. brucei* suppresses expression of abundant host proteins to promote MT-parasite transmission

Previous transcriptomic analyses on *G. m. morsitans* SGs reported seven protein families: (i) Tsal1/2; (ii) 5′Nuc/apyrase; (iii) ADA; (iv) TAg5 family-related proteins; (v) Glycine-glutamate (GE)-rich proteins; (vi) C-type lectins; and (vii) housekeeping genes [13, 41]. We identified these protein families with similar abundance profiles as reported across different tsetse species [11, 41–46]. Among these, the expression of *tag5*, *tsal1/2*, *tsgf1/2*, *5′-nuc* and *spg3* genes were previously reported to be decreased in parasitized flies [11]; these previous results agree with our results (see Table 1 and Additional file 1: Table S7). We however did not detect the anticoagulant tsetse thrombin inhibitor (TTI), echoing reports by Capello et al. [47] and Telleria et al. [13], who noted moderate down-regulation of TTI in parasitized SGs. It is notable that the differentially modulated proteins are involved in key processes such as blood feeding which, despite their high abundance, were downregulated in the parasitized SGs compared to unparasitized SGs (Table 1). Reduction in the expression of these proteins could reduce fly feeding performance, which in turn increases tsetse biting frequencies to achieve full engorgement [11]. One could conclude that an increase in biting frequencies promotes the competence of the tsetse vector in trypanosome transmission.

### *Trypanosoma b. brucei* induces upregulation of host proteins essential for parasite differentiation and survival

Cellular proliferation and homeostasis are important conditions for parasite survival. We identified several proteins, whose upregulation may be advantageous to *T. b. brucei* (see Table 2). For instance, CaMK is one of the most important regulators of stage-specific morphological differentiation of *Trypanosoma*, *Leishmania* and *Plasmodium* parasites [48]. Upregulation of Serp-2 is also notable owing to its central roles in ER stress, an important cue for *Plasmodium* parasites to switch to transmissible sexual stages in mosquitoes [49]. Further, one of the parasitic survival tactics is to alter intra-cellular locations, binding partners and/or functions of specific proteins and antagonizing other protein modifications. One of the quickest ways to achieve these changes is via sumoylation [50, 51], a posttranslational modification mediated by protein SUMO. Notable also is the upregulation of proteins in the family of the ubiquitous and highly conserved V-ATPases; at least eight different V-ATPase subunits were variously upregulated in parasitized SGs (see Additional file 1: Table S5). Mosquito cells over-expressing V-ATPases are reported to be preferentially invaded by *Plasmodium* parasites [52]; perhaps trypanosomes has a similar invasion preference for V-ATPase expression in SGs. Homeostasis-associated proteins such as ArgK are also important for parasite survival; dsRNA-mediated silencing of *ArgK* significantly reduced *Plasmodium* parasite loads in *Anopheles gambiae* [53]. In our study, ArgK was not only the most abundant protein (Table 1), but also amongst the upregulated proteins (see Additional file 1: Table S5).

### *Glossina m. morsitans* overexpresses immunity-related proteins in responses to SG parasitisation

Overcoming host immune responses is one of the most challenging parts of trypanosome life-cycle because immunity counter-defence provides a nutritious and equable environment. Lehane et al. [54] reported that out of the 68 putative immunity-related genes, 15 genes were actually overexpressed in the midguts of *T. b. brucei*-infected *G. m. morsitans.* Midgut expression of 12 of the 15 genes in *T. b. gambiense*-infected *G. p. gambiensis* was recently quantified by Hamidou et al. [55], who noted a time-dependent and variable high expression of five out of the 12 genes. Eight of these previously reported immune-related proteins were upregulated in parasitized SGs (see Table 3), a result in agreement with another transcriptome-based study on parasitized SGs of *G. m. morsitans* [13]. The only exemption was that only Serp-2 and CatB were significantly upregulated in parasitized SGs; CatB was not reported by Hamidou et al.*,* [55]. These differences potentially indicate tissue-specific differential expression patterns of these proteins, for instance in the midgut *versus* the SGs.

In addition to the above-mentioned previously reported tsetse immunity genes, we also identified other proteins implicated in the immunity of other insects (see Table 3). These included Rac/Rab GTPases (for immune surveillance against pathogens and parasites in *Drosophila* [56]), ubiquitin protein ligase (for *Drosophila* humoral systemic immune response [57]), a homolog to the Pro-Glu/polymorphic GC-rich repeat (PE-PGRS) protein (for antigenic variations in intracellular parasites [34]), Tsp42Ed and integrins (for activation of immune signaling *Plasmodium*-infected mosquitoes [53, 58]) and Drk (implicated in *Drosophila* hemocyte immunity [59]). Finally, loosely associated to insect immunity is amino acid metabolism; some of the upregulated proteins we identified are related to amino acid metabolism (see Table 1). However, a link between upregulation of proteins involved in amino acid metabolism and parasite infection in the SGs is unclear. This notwithstanding, it is rather obvious that following a blood meal, increase in amino acid metabolism is important for insect immune responses (via activation of proteolytic immunity cascades such as prophenoloxidase) and detoxification (removal of amino acid metabolites). Although likely to occur in the hemolymph, these processes are reportedly present in the SGs of locusts, leafhoppers and aphids [60]. Similar scenario cannot be totally ruled out in the case of tsetse immune responses to trypanosome infections in the SGs.

### *Trypanosoma b. brucei* controls the efficiency of protein turnover in the SGs of *T. b. brucei*

It is notable that > 20 % of the downregulated host-specific proteins in parasitized SGs were RPs. This modulation of RP expression is in agreement with recent transcriptome-based studies in the differential expression of the RP genes in *T. b. gambiense*-infected *G. p. gambiensis* midguts [61]. Downregulation of RPs implicates protein translational regulation to prepare mammalian-infective MT-parasites, or reflects the fly’s adaptive response to SG parasitisation. Notably, the downregulation of RPs appears to be accompanied by upregulation of at least 17 proteins related to the UPS pathway, suggesting that metacyclogenesis requires protein translation and/or degradation efficiency. Moreover, the importance of protein turnover/folding/modifications during parasite infections is underscored by differential modulations of the UDE homolog [62], TcP-1 components [63] and various molecular chaperones identified in the current study.

### *Trypanosoma b. brucei* induces upregulation of key regulators (host proteins) of diverse pathways

The identification of the significantly upregulated SUMO as one of the top interacting proteins in the predicted PPI network (Fig. 3) is interesting given its central roles in the regulation of various cellular processes including transcription, replication, chromosome segregation and DNA repair. SUMO is required for activation of the Ras/MAPK pathway in *Drosophila* S2 cells [64]; Drk is one of the essential components of the pathway. Additionally, the 14-3-3 protein family, which constitute a multitude of functionally diverse signalling proteins related to behaviour, is also a SUMO substrate. Sumoylation of 14-3-3 proteins results in modulation of diverse cellular processes, including cell cycle regulation, metabolism control, apoptosis and gene transcription control [65]. Furthermore, sumoylation and ubiquitin-proteasome systems are known to selectively modify the functions, sub-cellular locations and half-life of proteins in a very specific manner to maintain cellular homeostasis [66]. According to our PPI predictions (see Fig. 3 and Additional file 1: Table S12) sumoylation targets include several proteins of the TcP-1 complex, members of the 70-kDa heat shock protein family (Hsp70-4 and Hsp70-5), translation initiation factors and several enzymes (see Fig. 3).

On the other hand, members of the NADH-ubiquitin oxidoreductases (in our case the nuo-24 protein; significantly upregulated; Table 2) are important for energy metabolism and nucleotide synthesis; these proteins are also the initial enzymes in mitochondrial transport chain (mtECT). In fact, mtECT-related proteins were reported to be upregulated during the invasion of mosquito SGs by *Plasmodium* parasites [67]. Following a blood meal, GS (a key enzyme in glutamine biosynthesis) is upregulated in mosquito midguts [68], which agrees with the results shown in Additional file 1: Table S5. Glutamine is eventually used for production of chitin, which is also produced in other tissues including the SGs. Perhaps the upregulation of GS is related to chitin biosynthesis in *Glossina*, requiring modulation during parasite infection as can be observed in our PPI predictions, i.e. Serp-4 and ubiquitin-related proteins were first neighbours of GS (see Fig. 3). Finally, the central hub occupied by the V-ATPases in the predicted PPI network is noteworthy because these electrolyte/solute trans-epithelia transporters are critical in saliva secretion in other insects such as the blowflies [69]. *Trypanosoma. b. brucei* may therefore modulate the expression of V-ATPases to facilitate saliva-mediated transmission to the mammalian host, which possibly explains the upregulation of various V-ATPases (Additional file 1: Table S5). Taken together, the central hubs occupied by the significantly upregulated proteins in the predicted PPI network potentially indicate manipulations of diverse cellular processes in response to trypanosome infection. These processes require strict control of the participating proteins and enzymes.

### *Trypanosoma. b. brucei* MT-parasites express specific proteins in preparation to invade mammalian host

During metacyclogenesis, the MT-parasites re-acquire the VSG/ISG coat and enter the SG lumens in preparation for invading susceptible mammalian hosts [70]. Expressions of VSG and the VSG-shielded ISGs are activated in the SGs to mediate the parasite’s evasion of and/or escape the immune surveillance of the host, and successful establishment of mammalian infections [71, 72]. Trypanosomes have other VSG-shielded proteins or proteins that are ‘hidden’ within the flagellar pocket, where they mediate numerous host-parasite interactions. For instance BARPs are predominantly used by the parasites to attach to the SG plasma membranes and promote the release of VSGs [32, 73]. In fact, mutations of BARPs are reported to block cytokinesis in *T. b. brucei* [74]. Further, expression site-associated gene (ESAG) proteins are widely distributed amongst different *Trypanosoma* species albeit with low expression [72, 75], probably explaining the detection of only few ESAG variants in our study (see Table 4 and Additional file 1: Table S3). Notably, to enhance adaptive immunity, *T. brucei* switches the expression of VSGs with compatible ESAG variants [76]. Moreover, the identification of members of the diverse and rapidly evolving RHS family in our study is noteworthy (see Table 4). Since RHS proteins are localized in polymorphic subtelomic regions [77], they are likely to confer the MT-parasites with selective advantages (via antigenic variations). Additionally, detoxification is also important in parasite metabolism, which underscores the importance of the abundance of TXNs, members of the peroxiredoxin AhpC-type family (Table 4). Abundant in all *T. brucei* life stages, TXNS are crucial in nucleotide synthesis and adaptive defence (hyperoxide detoxification) [78, 79].

KMP-11 and CALP/SKCRP are well-conserved heterodimeric proteins with strict stage-specific expression patterns in kinetoplastids [80–82]. Stebeck et al. [82] reported that KM-11 is differentially expressed in various trypanosome life-cycle stages. CALP/SKCRP are abundantly expressed in mammalian-infective MT-parasites, and are involved in various cellular Ca^2+^-regulated processes such as signal transduction, differentiation, virulence, cytoskeletal rearrangement, and protein-membrane interactions with host structures [80, 83]. Out of the 18 (12 CALPs; six SKCRPs) family members known in *T. brucei* [81], we identified eight including CAP5.5 V, a variant of the insect stage-specific CAP5.5 (see Table 4). Finally, one of the uncharacterized proteins that we identified (C9ZWF1; Table 5) was homologous to the *T. grayi* calpain-like cysteine peptidase, an important protein in parasite cell cycle progression [84].

The interchange between the functionally and morphologically distinct forms of trypanosomatids (from epimastigotes to trypomastigotes) is accompanied by efficient regulation of gene expression, which is largely posttranslational [85]. Among other strategies, this regulation can be through the control of the expression of translation factors; translation of RPs is reduced during differentiation of trypanosomatids into the MT-parasites [86]. The possibility of this form of translational repression partially explains our detection of only few RPs (see Table 4), potentially because the MT-parasites are essentially growth-arrested. Our results echo studies on the on differential expression of RPs in the closely related Chagas disease causative agent, *T. cruzi* [86].

During active replication, trypanosomes heavily rely on membrane transporters for nutrient and metabolite salvage from the host. The main transporters include purine/pyrimidine transporters (trypanosomes are incapable of *de novo* purine synthesis) [87], hexose/sugar transporters (to meet parasite’s nutritional requirements) [88], amino acid transporters (for incorporation into proteins by the parasite) [89, 90], aquaporins (for osmotic protection and metabolism) [91, 92] and fatty acids transporters (for preservation of organelle vitality and expansion of vacuole sizes to the accommodate growing parasite progenies) [93]. *Trypanosoma brucei* contains a repertoire of 305 transporters clustered in 23 families [94]. We identified only three transporters, all with low abundances (see Table 4). Potentially, the growth-arrested MT-parasites may not require expression of many transporters; expression of the transporter genes is probably activated in the next life-cycle stage, i.e. proliferating bloodstream forms in the mammalian host.

### Potential approaches to enhance trypanosome refractoriness in tsetse

Factors such as the lack of effective vaccines, resistance to pesticides/drugs and inefficient public health infrastructures make African trypanosomosis increasingly important in sub-Saharan Africa. The fact that sterile males are still competent trypanosome vectors reduces the efficacy of tsetse and trypanosomosis control via the SIT programs. One of the strategies to solve this problem is to enhance the natural trypanosome refractoriness or reduce the competence of the tsetse vector. This can be achieved by identifying candidate genes and creating transgenic sterile males that are incapable of transmitting trypanosomes to mammalian hosts. Ideally, the transgenic sterile males released into wild tsetse populations could eventually block trypanosome transmission. This would be important especially in the event that the areas into which these males are released have active trypanosome circulation.

Although a proteomic approach is far better (in protein identification and observed quantitative expression changes) than a transcriptomic approach, the former approach is often not precise enough to directly make valid biological inferences, especially due to the post-translational modifications that play important roles in protein functions. Nevertheless, our proteomics and pathway analyses are important milestones in the identification and characterization of SG proteins that potentially contribute to the trypanosome infection status (refractoriness) in the tsetse vector. Having confirmed the protein abundances and expression patterns (parasitized *vs* unparasitized SGs), we are now designing bioassays to functionally appraise the biological consequences (by immunoblotting, qPCR, RNAi gene silencing) of the protein level changes of some of the proteins identified in our proteomics data sets. After validation, the next objective is to use modern tools such as the *piggyBac* transposon or CRISPR/Cas9 systems [95], or paratransgenesis [96] to enhance trypanosome refractoriness of the sterile males that are used in SIT programs. Previous studies have provided proof of principle that these methods are feasible in creation of parasite-refractory insect vectors. For instance, using the *piggyBac* system, Ito et al. [97] created *Plasmodium*-refractory anopheline mosquitoes by over-expressing a blood-inducible anti-plasmodium gene (salivary gland/midgut-binding peptide 1; SM1) in a tissue-specific manner. Recently, Gantz et al. [98] used the CRISPR/Cas9 system to create *Plasmodium*-resistant mosquitoes by expression of blood-inducible and tissue-specific single chain variable fragment antibodies (scFvs) against *Plasmodium* proteins. In the case of tsetse flies, De Vooght et al. [96] demonstrated that transformed *Sodalis* can secrete significant amounts of functional Nanobodies against *Trypanosoma* VSG epitopes. The concept of paratransgenesis has also been demonstrated in the control of *Leishmania* transmission by sand flies [99]. Taken together, our data take us a step closer towards improved anti-vector methods against tsetse and African trypanosomiasis. We have made significant progress towards this direction in that the Joint FAO/IAEA Division of Nuclear Techniques in Food and Agriculture has brought together research experts in different disciplines in a collaborative coordinated research project to explore ways to enhance trypanosome refractoriness in tsetse [2].

## Conclusions

Our data suggest that *T. b. brucei* modifies SG protein composition and functions (suppression of abundant SG proteins) and induces SG cellular proliferation (upregulation of immunity, stress, homeostasis and translatome-related proteins). Further, the repertoire of *T. b. brucei*-specific proteins largely consisted of proteins reminiscent of non-replicative MT-parasites (suppression of RPs and transporters), and proteins critical for preparing the trypomastigotes for invasion and evasion of mammalian host immune responses (over-representation of immunity, signal transduction and virulence-related proteins). In response to *T. b. brucei* infection of *G. m. morsitans* SGs, divergent cellular processes appear to be manipulated via modulations of proteins involved in various pathways, which is accompanied by modulations of proteins involved in the control of protein turnover*.* Similar to the tsetse midgut barrier, SG micro-environment is a critical bottleneck with key determinants to *T. b. brucei* life-cycle transitions. Our proteomic data provide evidence that these genes are not only transcribed (as evidenced by previous transcriptome-based studies), but are also translated into (potentially functional) proteins. These proteins, especially the immunity-related proteins, present an attractive platform to enhance trypanosome refractoriness as an anti-vector strategy to control tsetse and African trypanosomosis. However, as we have discussed above, we are aware of the need to experimentally validate our proteomics data, a process which is now on-going.

## Abbreviations

5′ Nuc, 5′-nucleotidase-related saliva protein; ABC, ABC ammonium bicarbonate; ACN, acetonitrile; ADA, salivary adenosine deaminase; ADGF, adenosine deaminase growth factor; AhpC, alkyl hydroperoxide reductase; AIR9, auxin-induced in root cultures 9; APS, bifunctional ATP/sulfurylase-adenosine 5′-phosphosulfate; ArgK, arginine kinase; AspAT, aspartate aminotransferase; AUH, methylglutaconyl-CoA hydratase; BARP, bloodstream stage alanine-rich proteins; BLAST, basic local alignment search tool; BP, biological process; CALP, calpain; CaMK, Ca2+/calmodulin-dependent protein kinase; CatB, cathepsin B-like cysteine proteinase; CBB, colloidal brilliant blue; CC, cellular component; CRISPR/Cas9, Clustered Regularly Interspaced Short Palindromic Repeat/CRISPR associated protein 9; DBT, dihydrolipoamide transacylase α-ketoacid dehydrogenase; Drk, downstream of receptor kinase; ESAG, expression site-associated gene; FAZ, flagellar attachment zone protein; FDR, false discovery rate; GE-rich, glycine-glutamate-rich protein; gGAPDH, glycosomal glyceraldehayde-3-phosphate dehydrogenase; GO, gene ontology; GPI, glycosylphosphatidyl inositol; GS, glutamine synthetase; GST1, glutathione S-transferase 1; iBAQ, intensity-based absolute quantitation; IID, insect innate immunity database; IPR, iron regulatory protein; ISG, invariant surface glycoprotein; IVD, isovaleryl-CoA dehydrogenase; KMP-11, 11-kDa kinetoplastid membrane protein-11; LC-MS/MS, liquid chromatography coupled to electrospray and tandem mass spectrometry; LFQ, label-free quantification; MF, molecular function; MT, mammalian-infective metacyclic trypomastigote; mtECT, mitochondrial transport chain; MTHF-folD, bifunctional methylene-tetrahydrofolate dehydrogenase; NOS, nitric oxide synthase; nuo-24, mitochondrial NADH-ubiquinone oxidoreductase; OSM3, osmotic avoidance abnormal protei3; P5CDH, δ-1-pyrroline-5-carboxylate dehydrogenase; PFR, paraflagellar rod protein; PPI, protein-protein interaction; PSMA/PSMB, proteasome regulatory complex proteins α-/β-types; Ras-MAPK, Ras-mitogen-activated protein kinase; RH, relative humidity; RHS, retrotransposon hot spot protein; RpS27A, ubiquitin-40S ribosomal protein S27a fusion protein; scFvs, single chain variable fragment antibodies; SDS-PAGE, sodium dodecyl sulphate polyacrylamide gel electrophoresis; Serp-2, stress-associated endoplasmic reticulum protein 2; SG, salivary gland; Sgp3, 5′ nucleotidase-related SG protein 3; SIT, sterile insect technique; SKCRP, small kinetoplastid calpain-related protein; SUMO, small ubiquitin-related modifier 3; TAg5, antigen-5-related allergen; TcP-1, Chaperonin containing t-complex polypeptide 1; TRAP, translocon-associated complex protein; TrxP, thioredoxin peroxidases; Tsal1/2, tsetse salivary gland proteins 1 and 2; Tsp, tetraspanin; TXN, tryparedoxin; uBA1, ubiquitin activating enzyme; uCHL, ubiquitin C-terminal hydrolase; UDE, uracil-DNA degrading factor-like protein; UPS, ubiquitin-proteasome system; V-ATPases, vacuolar ATPases; VSG, variant surface glycoprotein

## Additional file

Additional file 1: Table S1. 523 non-redundant protein groups identified by LC-MS/MS. **Table S2.** 362 protein groups specific to the host vector. **Table S3.** 158 protein groups specific to the parasite identified in parasitized SG of *G. m. morsitans*. **Table S4.** Four bacterial endosymbiont proteins identified in the *G. m. morsitans* by LC-MS/MS. **Table S5.** 276 protein groups upregulated in the parasitized SGs of *G. m. morsitans*. **Table S6.** 32 protein groups significantly upregulated in the parasitized SG of *G. m. morsitans*. **Table S7.** 81 protein groups downregulated in parasitized SGs. **Table S8.** Four host proteins not affected by parasite infection. **Table S9.** Distribution of GO terms associated with biological processes. **Table S10.** GO terms associated with molecular functions. **Table S11.** GO terms associated with cellular compartments. **Table S12.** 225 protein pairs with significant interacting probabilities (≥0.2) determined by FpClass. (XLSX 352 kb)
